# Myocardial injury in patients with acute ischemic stroke: Prevalence and types of triggers of myocardial demand ischemia

**DOI:** 10.1093/esj/23969873251346008

**Published:** 2026-01-01

**Authors:** Helena Stengl, Sophie Böhme, Oskar Richter, Simon Hellwig, Markus G Klammer, Ramanan Ganeshan, Laura Reimann, Heinrich J Audebert, Wolfram Doehner, Christian H Nolte, Matthias Endres, Jan F Scheitz

**Affiliations:** Department of Neurology with Experimental Neurology, Charité – Universitätsmedizin Berlin, Berlin, Germany; Center for Stroke Research Berlin (CSB), Charité – Universitätsmedizin Berlin, Berlin, Germany; German Center for Neurodegenerative Diseases (DZNE), Partner Site, Berlin, Germany; Department of Neurology with Experimental Neurology, Charité – Universitätsmedizin Berlin, Berlin, Germany; Center for Stroke Research Berlin (CSB), Charité – Universitätsmedizin Berlin, Berlin, Germany; Department of Neurology with Experimental Neurology, Charité – Universitätsmedizin Berlin, Berlin, Germany; Center for Stroke Research Berlin (CSB), Charité – Universitätsmedizin Berlin, Berlin, Germany; Department of Neurology with Experimental Neurology, Charité – Universitätsmedizin Berlin, Berlin, Germany; Center for Stroke Research Berlin (CSB), Charité – Universitätsmedizin Berlin, Berlin, Germany; Berlin Institute of Health (BIH) at Charité – Universitätsmedizin Berlin, Berlin, Germany; Department of Neurology with Experimental Neurology, Charité – Universitätsmedizin Berlin, Berlin, Germany; Center for Stroke Research Berlin (CSB), Charité – Universitätsmedizin Berlin, Berlin, Germany; Center for Stroke Research Berlin (CSB), Charité – Universitätsmedizin Berlin, Berlin, Germany; Department of Neurology, Asklepios Fachklinikum Teupitz, Teupitz, Germany; Department of Neurology with Experimental Neurology, Charité – Universitätsmedizin Berlin, Berlin, Germany; Center for Stroke Research Berlin (CSB), Charité – Universitätsmedizin Berlin, Berlin, Germany; German Center for Neurodegenerative Diseases (DZNE), Partner Site, Berlin, Germany; Department of Neurology with Experimental Neurology, Charité – Universitätsmedizin Berlin, Berlin, Germany; Center for Stroke Research Berlin (CSB), Charité – Universitätsmedizin Berlin, Berlin, Germany; Center for Stroke Research Berlin (CSB), Charité – Universitätsmedizin Berlin, Berlin, Germany; Berlin Institute of Health (BIH) at Charité – Universitätsmedizin Berlin, Berlin, Germany; Department of Cardiology (CVK), Deutsches Herzzentrum der Charité, Berlin, Germany; German Centre for Cardiovascular Research (DZHK), Partner Site, Berlin, Germany; Department of Neurology with Experimental Neurology, Charité – Universitätsmedizin Berlin, Berlin, Germany; Center for Stroke Research Berlin (CSB), Charité – Universitätsmedizin Berlin, Berlin, Germany; Berlin Institute of Health (BIH) at Charité – Universitätsmedizin Berlin, Berlin, Germany; German Centre for Cardiovascular Research (DZHK), Partner Site, Berlin, Germany; Department of Neurology with Experimental Neurology, Charité – Universitätsmedizin Berlin, Berlin, Germany; Center for Stroke Research Berlin (CSB), Charité – Universitätsmedizin Berlin, Berlin, Germany; German Center for Neurodegenerative Diseases (DZNE), Partner Site, Berlin, Germany; Berlin Institute of Health (BIH) at Charité – Universitätsmedizin Berlin, Berlin, Germany; German Centre for Cardiovascular Research (DZHK), Partner Site, Berlin, Germany; Department of Neurology with Experimental Neurology, Charité – Universitätsmedizin Berlin, Berlin, Germany; Center for Stroke Research Berlin (CSB), Charité – Universitätsmedizin Berlin, Berlin, Germany; Berlin Institute of Health (BIH) at Charité – Universitätsmedizin Berlin, Berlin, Germany; German Centre for Cardiovascular Research (DZHK), Partner Site, Berlin, Germany

**Keywords:** Ischemic stroke, Stroke-Heart Syndrome, acute myocardial injury, demand ischemia, type 2 MI, high-sensitivity cardiac troponin T, 4th UDMI, central autonomic network%

## Abstract

**Introduction:**

Acute myocardial injury occurs in about every fourth patient in the early phase after ischemic stroke. It may be caused by an imbalance between myocardial oxygen supply and demand, potentially leading to type 2 myocardial infarction (MI). However, little is known about the prevalence of potential triggers of such demand ischemia in acute stroke.

**Patients and methods:**

Consecutive patients with and without post-stroke acute myocardial injury (elevated high-sensitivity cardiac troponin T [hs-cTnT] levels with a rise/fall >20%) were matched for age and sex and retrospectively screened for presence of predefined triggering conditions of myocardial demand ischemia and fulfillment of diagnostic criteria for acute MI.

**Results:**

Among 508 stroke patients analyzed (median age 81 [73–86] years, 52% female), predefined potential triggers of demand ischemia were present in 107/254 (42%) patients with acute myocardial injury and in 61/254 (24%) matched controls (adjusted OR 2.30, 95%CI 1.51–3.52, *p* < 0.001). Patients with a trigger were older, more often female, had more severe strokes, and more often insular cortex involvement. The most prevalent triggers were respiratory failure, sustained hypertension, supraventricular tachyarrhythmia, and hemodynamic shock. MI criteria were fulfilled in 44/254 (17%) patients with acute myocardial injury including 27/44 (61.4%) with a trigger of demand ischemia (i.e. suspected type 2 MI).

**Conclusions:**

Conditions triggering a myocardial oxygen demand/supply mismatch are highly prevalent in patients with acute myocardial injury detected after stroke, notably in those fulfilling the criteria of acute MI. Stroke-specific aspects such as stroke severity or lesion location may play a role in the development of such triggers.

## Graphical Abstract

**Graphical abstract:**
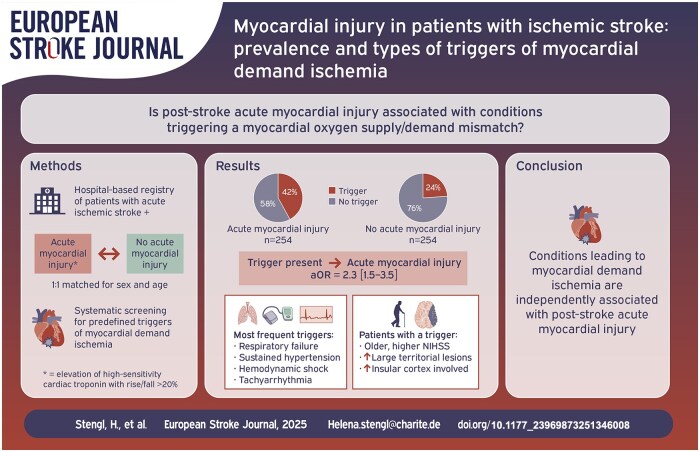

## Background

Acute myocardial injury defined as elevated cardiac troponin levels above the upper reference limit (URL) with a rise or fall pattern >20% in consecutive measurements occurs in up to one out of four patients with acute ischemic stroke.^1,2^ It represents one aspect of several possible cardiac complications after stroke that have been summarized under the conceptual framework of the so-called “stroke-heart syndrome” (SHS).^3–5^ Interpretation of troponin elevation in the acute setting of stroke remains challenging for the treating physician. However, it is important to identify stroke patients in need of further cardiac evaluation, given the higher mortality and worse functional outcome associated with acute myocardial injury.^2,6^

**Figure 1.**
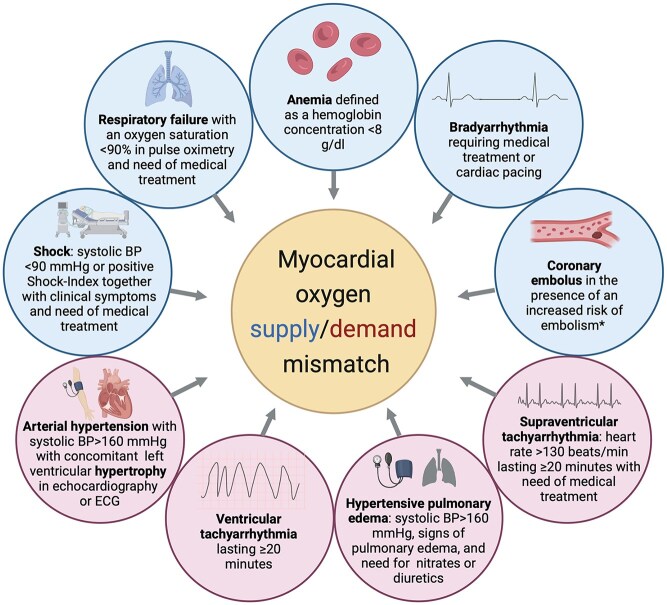
Predefined triggers of myocardial oxygen supply/demand mismatch. BP: blood pressure; ECG: electrocardiogram. *For further information on predefined triggering factors see Supplemental Table 1 online. Criteria based on previously published cut-offs from Saaby et al.^11^ Image created using biorender.

The underlying pathophysiological mechanisms of troponin elevation after stroke are still incompletely understood.^3,4^ In comparison to patients presenting with acute coronary syndrome, stroke patients are less likely to have atherothrombotic coronary lesions (type 1 myocardial infarction [MI]) in coronary angiography despite similar troponin levels.^7,8^ It has been suggested though, that myocardial oxygen supply/demand mismatch (as in type 2 MI) may be a relevant mechanistic pathway leading to myocardial damage in the context of acute stroke.^3,4^ Myocardial demand ischemia can be triggered by several mechanisms causing reduced myocardial oxygenation (e.g. anemia, respiratory failure, hypotension, or bradyarrhythmia) or situations of increased myocardial oxygen demand as for instance in severe hypertension or tachycardia (see Figure 1).^1,9–12^

### Aims

At present, the role of myocardial demand ischemia in the context of troponin elevation after stroke remains poorly understood. Therefore, we aimed to assess the prevalence, types, and characteristics of potential triggering factors of myocardial oxygen supply/demand mismatch in stroke patients with acute myocardial injury compared to those without. Subsequently, we aimed to determine how often criteria of acute MI according to the 4^th^ Universal definition of myocardial infarction (4^th^ UDMI) are met in stroke patients with acute myocardial injury and accompanying presence or absence of a trigger of demand ischemia.^1^

## Methods

### Study population

A detailed description of the studied population has been published earlier.^2,13^ Briefly, all consecutive patients admitted to the Stroke Unit of a tertiary care hospital were prospectively screened for inclusion in the CORONA-IS study (NCT03892226). The CORONA-IS study was approved by the local Ethics Committee of the Charité–Universitätsmedizin Berlin (EA4/123/18) and a comprehensive study protocol has been published previously.^14^

All patients from the screening database during the screening period between 01/2019 and 12/2020 with imaging confirmed acute ischemic stroke, hospital admission within 48 h of stroke symptom onset and repeated measurements of high-sensitivity cardiac Troponin-T (hs-cTnT; assay characteristics: Roche Elecsys^®^, Gen 5; 99th percentile URL = 14 ng/L) within the first 2 days of admission were eligible for the present analysis. Presence of acute myocardial injury was defined as at least one troponin value above the URL with a rise/fall of >20% in repeated measurements according to the 4^th^ UDMI.^1,14^ All patients with acute myocardial injury were 1:1 matched for age and sex to control patients without acute myocardial injury (i.e. normal hs-cTnT values or elevated values but without a rise or fall >20% in repeated measurements).

### Data collection

Patients’ demographics, cardiovascular risk factors, information regarding stroke symptom onset and in-hospital treatments were prospectively collected as described earlier.^2,13^ For the present analysis, we retrospectively gathered further clinical information from routine clinical records. This included cardiac comorbidities, cardiac diagnostics, routine blood tests, clinical complications, and stroke lesion characteristics (lesion side and pattern, presence of large vessel occlusion, space occupying edema, and insular cortex involvement). Furthermore, vital parameters were extracted from Stroke Unit monitoring records including blood pressure, heart rate, oxygen saturation, breathing rate and body temperature. Stroke severity was assessed via National Institutes of Health Stroke Scale (NIHSS) and functional status via modified Rankin Scale (mRS). In accordance with the laws and regulations of the Federal State of Berlin (§25 Landeskrankenhausgesetz), written informed consent is not required for a retrospective anonymized analysis of routine medical data obtained during the patients’ hospitalization within the own department.

All patient records were examined for presence of a predefined potential trigger of myocardial oxygen supply/demand mismatch (i.e. demand ischemia) which represents the pathological mechanism of type 2 MI (see Figure 1).

Definitions of these triggering conditions were based on previously published criteria applied in studies of patients with suspected MI.^11,15^ Specific cut-off values and applied modifications to account for differences in the analyzed stroke population can be found in the Supplemental Table 1 online. The analysis of vital parameters and presence of a potential trigger of demand ischemia was conducted for the period from hospital admission until the second hs-cTnT measurement on the following day to be considered relevant for the development of acute myocardial injury.

**Figure 2.**
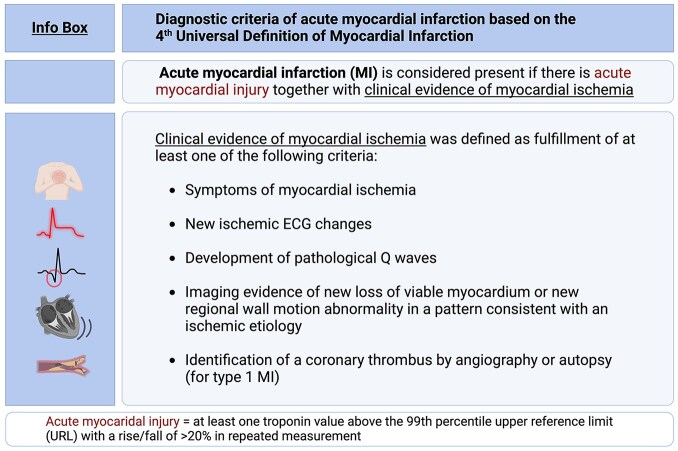
Info box: Diagnostic criteria of acute myocardial infarction based on the 4th Universal Definition of Myocardial Infarction (Thygesen, Alpert and Jaffe, 2019).

Patient records were systematically analyzed for fulfillment of the diagnostic criteria of acute MI specified by 4^th^ UDMI.^1^ Accordingly, acute MI was considered present if there was acute myocardial injury together with clinical evidence of myocardial ischemia (see Figure 2; Info Box).

### Statistical analysis

Categorical variables are presented as frequencies. Based on their distribution, continuous variables are presented as mean ± standard deviation (SD) or median with interquartile range (IQR). Group comparisons were conducted using independent samples t-tests, Wilcoxon-Mann-Whitney *U* tests, and Chi-square or Fisher’s exact tests, as appropriate. Unadjusted and adjusted binomial logistic regression analysis was performed to calculate odds ratios (OR) and 95% confidence interval (CI).

To analyze the association of the presence of a potential trigger of cardiac demand ischemia with acute myocardial injury, adjustments were made for possible risk factors of hs-cTnT elevation in stroke.^2,6,16^ These included NIHSS at admission, time from stroke onset to admission, history of chronic kidney disease, diabetes, atrial fibrillation, coronary artery disease (CAD), congestive heart failure, and cancer. As the cohort was matched 1:1 based on age and sex, these two variables were not included in the model for this outcome of interest.

Characteristics and outcomes of all patients with and without a trigger – irrespective of the presence of acute myocardial injury – were compared as well as the troponin values between patients with acute myocardial injury and with and without fulfillment of criteria for acute MI according to 4th UDMI.^1^ All tests were two-sided and values of *p* < 0.05 were considered statistically significant. Statistical analyses were performed using IBM SPSS (SPSS, Inc., Chicago, IL, version 29.0) and RStudio (RStudio: Integrated Development for R. RStudio, PBC, Boston, version 2024.09).

## Results

### Patient characteristics

**Figure 3.**
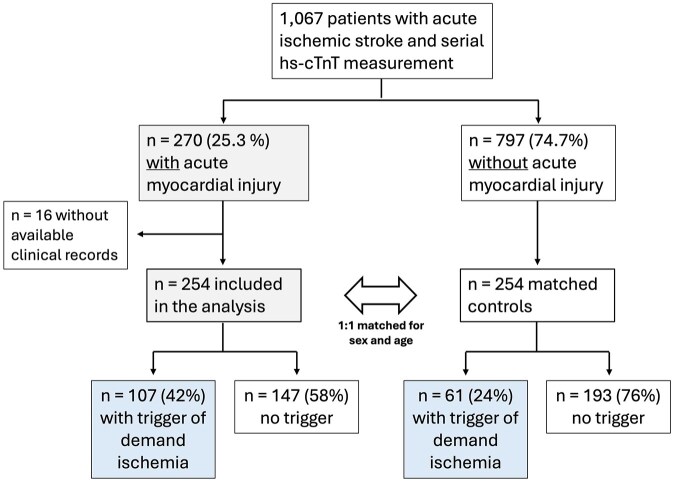
Flowchart of included patients and prevalence of triggers of myocardial oxygen supply/demand mismatch.

Out of 1067 consecutive patients with neuroimaging confirmed ischemic stroke and repeated hs-cTnT measurement screened from 01/2019 until 12/2020, a total of 270 patients showed acute myocardial injury (as published earlier).^2^ Of these, 16 patients had no available hospital records for further analysis and therefore had to be excluded. There were no statistically significant differences in demographic or clinical data such as age, sex, NIHSS, rate of revascularization therapy, or cardiovascular comorbidities between included and excluded patients (all *p* > 0.05). The remaining 254 patients with acute myocardial injury were matched 1:1 for age and sex to patients without acute myocardial injury, resulting in a cohort size of 508 patients in total (see Figure 3). Median age was 81 years (73–86), 52% were female and median NIHSS upon admission was 4 [2–10], see Table 1.

**Table 1.** Baseline characteristics and comparison of stroke patients with vs. without acute myocardial injury.

Baseline variables	Total cohort *n* = 508	Acute myocardial injury *n* = 254 (50%)	No acute myocardial injury *n* = 254 (50%)	*p* Value
Demographic data				
Median age, years	81 [73–86]	81 [73–86]	81 [73–85]	1.0
Sex female	266 (52.4)	133 (52.4)	133 (52.4)	1.0
Comorbidities				
Hypertension	402 (79.1)	207 (81.5)	195 (76.8)	0.19
Previously known atrial fibrillation	107 (21.1)	58 (22.8)	49 (19.3)	0.33
AFDAS	60 (11.8)	39 (15.4)	21(8.3)	0.013
Coronary artery disease	110 (21.7)	56 (22.0)	54 (21.3)	0.83
Prior myocardial infarction	48 (9.4)	26 (10.4)	22 (8.7)	0.52
Prior ischemic stroke	166 (32.7)	86 (33.9)	80 (31.5)	0.57
Diabetes mellitus	127 (25.0)	62 (24.4)	65 (25.6)	0.76
Dyslipidemia	249 (49.0)	123 (48.4)	126 (49.6)	0.79
Chronic kidney disease	115 (22.6)	71 (28.0)	44 (17.3)	0.004
Chronic heart failure	44 (8.7)	27 (10.6)	17 (6.7)	0.12
Malignancy	82 (16.1)	51 (20.1)	31(12.2)	0.016
Stroke				
NIHSS at admission	4 [2–10]	6 [2–13]	3 [2–7]	<0.001
Revascularization therapy (IVT and/or EVT)	197 (38.8)	116 (45.7)	81 (31.9)	0.001
Stroke etiology cardioembolic	194 (38.2)	106 (41.7)	88 (34.6)	0.10
Large vessel occlusion	111 (21.9)	71 (28.0)	40 (15.7)	<0.001
Insular cortex affected	113 (22.2)	75 (29.5)	38 (15.0)	<0.001
Space occupying lesion^a^	26 (5.1)	20 (7.9)	6 (2.4)	0.005
Territorial infarction	73 (14.4)	40 (15.7)	33 (13.0)	0.38
Hemorrhagic transformation	71 (14.0)	47 (18.5)	24 (9.4)	0.003
Complications				
Infection^b^	81 (15.9)	49 (19.3)	32 (12.6)	0.04
Delirium^b^	29 (5.7)	21 (8.3)	8 (3.1)	0.013
Length of stay in days [IQR]	5 [3–8]	6 [3–9]	4 [3–7]	<0.001
Stroke outcomes				
NIHSS at discharge	2 [0–6]	3 [0–8]	1 [0–3]	<0.001
mRS at discharge	3 [1–4]	4 [1–5]	2 [1–4]	<0.001
In-hospital mortality	41 (7.3)	31 (12.2)	10 (3.9)	0.001

AFDAS: atrial fibrillation detected after stroke; EVT: endovascular thrombectomy; IVT: intravenous thrombolysis; mRS: modified Rankin Scale score; NIHSS: National Institutes of Health Stroke Scale.

Values are presented as median with [interquartile range] or absolute numbers *n* with (frequencies %).

^a^Defined as expansion in volume of infarcted brain tissue with displacement of normal neural structures according to patients’ radiology report.

^b^Infection or delirium diagnosed within the first 2 days of hospital admission.

### Vital signs and potential triggers of demand ischemia

Compared to patients without, patients with acute myocardial injury showed more pronounced deviations of vital signs during the first day of hospitalization. These included highest and lowest systolic blood pressure, highest and lowest heart rate, highest breathing rate, and lowest oxygen saturation in pulse oximetry (see Supplemental Table 2 online)

**Figure 4.**
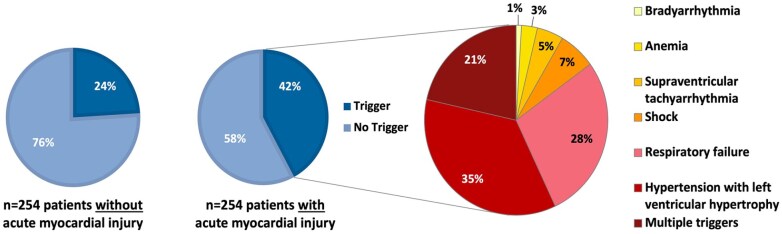
Prevalence and types of predefined triggers of myocardial oxygen supply/demand mismatch in stroke patients with and without acute myocardial injury. Multiple triggers: presence of ⩾2 triggers.

A total of 168 (33%) of all patients had evidence of a triggering condition of myocardial oxygen supply/demand mismatch. Overall, the most prevalent conditions were arterial hypertension (>160 mmHg) with concomitant left ventricular hypertrophy, respiratory insufficiency, and shock (see Supplemental Table 2 online). Prevalence of at least one predefined potential trigger of demand ischemia was higher in patients with acute myocardial injury (*n* = 107, 42.1%) than in those without (*n* = 61, 24%, *p* < 0.001; see Figure 4). A total of *n* = 23 (9.1%) patients with acute myocardial injury presented with multiple triggers compared to *n* = 7 (2.8%) in the control group. In patients with multiple triggering conditions, respiratory insufficiency, arterial hypertension with concomitant left ventricular hypertrophy, and sustained supraventricular tachyarrhythmia with need of medical treatment (e.g. beta-blocker, digitalis) were most frequent.

Overall, patients with triggers of myocardial oxygen supply/demand mismatch were older, more often female and had higher prevalence of cardiac comorbidities including atrial fibrillation (AF), CAD, or chronic heart failure, and higher rates of in-hospital mortality. Furthermore, they presented with higher stroke severity upon hospital admission and discharge, more frequently had insular cortex involvement, large territorial infarctions, and space occupying lesions (all *p* < 0.05, see Table 2).

**Table 2.** Baseline characteristics of patients with and without a predefined trigger of myocardial oxygen supply/demand mismatch.

Baseline variables	Presence of a trigger *n* = 168 (33.1%)	Absence of a trigger *n* = 340 (66.9%)	*p* Value
Age, years	83 [76–87]	80 [72–85]	0.001
Sex, female	108 (64.3)	158 (46.5)	<0.001
Acute myocardial injury	107 (63.7)	147 (43.2)	<0.001
Peak hs-cTnT, ng/L	36 [22–90]	25 [14–47]	<0.001
Onset to admission, hours	10.8 [2.2–20.3]	5.4 [1.8–13.5]	0.002
Comorbidities			
Arterial hypertension	140 (83.3)	262 (77.1)	0.10
Previously known atrial fibrillation	49 (29.2)	58 (17.1)	0.002
AFDAS	25 (14.9)	35 (10.3)	0.13
Coronary artery disease	47 (28.0)	63 (18.5)	0.02
Prior myocardial infarction	19 (11.3)	29 (8.6)	0.33
Prior stroke	52 (31.0)	114 (33.5)	0.56
Diabetes mellitus	46 (27.4)	81 (23.8)	0.38
Dyslipidemia	78 (46.4)	171 (50.3)	0.41
Chronic kidney disease	43 (25.6)	72 (21.2)	0.26
Chronic heart failure	24 (14.3)	20 (5.9)	0.002
Stroke			
Stroke etiology cardioembolic	85 (50.6)	109 (32.1)	<0.001
NIHSS at admission	6 [2–15]	3 [2–8]	<0.001
Major stroke [NIHSS ⩾ 5]	103 (62.0)	138 (41.1)	<0.001
Space occupying lesion	15 (8.9)	11 (3.2)	0.006
Territorial infarction	37 (22.0)	36 (10.6)	<0.001
Large vessel occlusion	46 (27.4)	65 (19.1)	0.03
Insular cortex affected	51 (30.4)	62 (18.2)	0.002
Revascularization treatment (IVT and/or EVT)	64 (38.1)	133 (39.1)	0.82
Outcome			
NIHSS at discharge	3 [1–10]	1 [0–4]	<0.001
mRS at discharge	4 [2–5]	2 [1–4]	<0.001
In-hospital mortality	25 (14.9)	16 (4.7)	<0.001

AFDAS: atrial fibrillation detected after stroke, EVT: endovascular thrombectomy, IVT: intravenous thrombolysis, mRS: modified Rankin Scale score, NIHSS: National Institutes of Health Stroke Scale.

Values are presented as median with [interquartile range] and absolute numbers with (frequency %).

Presence of a potential trigger was associated with acute myocardial injury in univariate logistic regression analysis (OR: 2.30, 95% CI: 1.57–3.37, *p* < 0.001) and after multiple adjustment (aOR: 2.30, 95% CI: 1.51–3.52, *p* < 0.001).

### Diagnostic criteria for MI in patients with acute myocardial injury

All patients’ clinical records were analyzed for fulfilment of criteria for myocardial ischemia based on clinical symptoms, ECG and further invasive and non-invasive cardiac imaging. Coronary angiography was performed in a total of *n* = 23/254 (9%), transthoracic echocardiography in *n* = 66/254 (26%) and transesophageal echocardiography in *n* = 45/254 (17.7%) patients with acute myocardial injury in this acute setting. Based on the available routine cardiac diagnostics, the diagnostic criteria for acute MI according to the 4th UDMI were fulfilled in 44 (17%) of 254 patients with acute myocardial injury. Of these 44 patients, *n* = 23 patients fulfilled multiple criteria of myocardial ischemia and *n* = 21 patients one additional criterion (*n* = 12 ischemic ECG changes, *n* = 3 clinical symptoms of myocardial ischemia, and *n* = 6 new regional wall motion abnormalities in cardiac imaging). Among patients with acute myocardial injury and a potential trigger (*n* = 107), 27 (25%) met the criteria for acute MI (i.e. suspected type 2 MI). Of patients with acute myocardial injury but without a trigger (*n* = 147), acute MI was identified in 17 patients (11.6%). In the remaining 210/254 patients no evidence of clinical signs, or ECG changes indicative of myocardial ischemia were detected, and in case of available cardiac imaging, no new wall motion abnormalities were found. Consequently, no diagnosis of acute MI was made by the treating stroke physician or consulting cardiologist during the acute in-hospital setting in these 210 patients.

**Figure 5.**
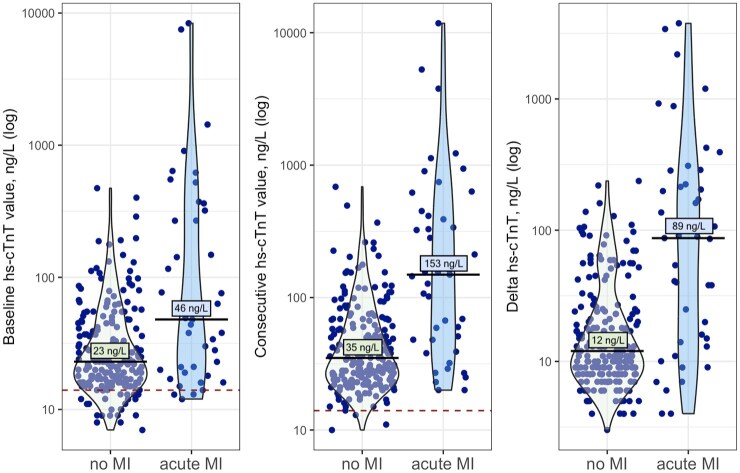
Median baseline, median consecutive high-sensitivity troponin T (hs-cTnT) values, and median difference between both values (delta) for stroke patients with acute myocardial injury without and with evidence for acute myocardial infarction (MI). Hs-cTnT values presented in logarithmic scale; red dashed line indicating 99th percentile URL (14 ng/L) of hs-cTnT. Baseline hs-cTnT values were measured at a median time of 7 h [2–18] and consecutive values at 24 h [16–35] after stroke symptom onset.

When analyzing all patients with acute myocardial injury, those with evidence of acute MI showed statistically significant higher median hs-cTnT values in baseline measurement than those without acute MI (46 ng/L [19–307] vs 23 ng/L [16–42]). This was also found in median hs-cTnT levels in consecutive measurement (153 ng/L [44–440] vs 35 ng/L [25–61]) as well as in larger median differences between both values (median delta 89 ng/L [14–270] vs 12 ng/L [8–24], all *p* < 0.001; see Figure 5).

## Discussion

Our analysis provides the following main findings relevant for the clinical care of acute ischemic stroke patients presenting with acute myocardial injury: First, the occurrence of predefined potential triggers of myocardial demand ischemia were almost 2-times more frequent in stroke patients with acute myocardial injury than in matched patients without. Second, the most prevalent triggering conditions were respiratory failure, elevated blood pressure values in patients with left ventricular hypertrophy, hemodynamic shock, and sustained supraventricular tachyarrhythmia. Third, stroke specific aspects such as higher stroke severity and strategic stroke lesions affecting the insula were more frequent in patients with a predefined trigger. Finally, among patients with acute myocardial injury and evidence of a trigger, approximately every fourth patient fulfilled the criteria for acute MI according to 4^th^ UDMI.

In the present study, predefined triggering conditions for demand ischemia in the early phase after stroke were associated with the development of acute myocardial injury. Since we specifically screened patients’ medical records for presence of a trigger between admission and second hs-cTnT measurement, we can reasonably assume that the triggering condition aligns with the time course of acute myocardial injury development, rather than occurring afterward. When comparing patients with a predefined potential trigger to those without, we found several stroke-specific differences such as stroke severity, rate of large vessel occlusion, large territorial- or space occupying lesions, and involvement of the insular cortex. These findings support the hypothesis that neurocardiogenic mechanisms play an important role in the development of post-stroke acute myocardial injury, and possibly other manifestations of the stroke-heart syndrome.^3–5,17^

Evidence suggests that stroke-related disruption of the central autonomic network resulting in an imbalance of the sympathetic nervous system represents a “stress-test” for the heart.^4^ Cardiac responses – such as tachycardia, blood pressure dysregulation and microvascular dysfunction – may act as key drivers for myocardial oxygen supply/demand mismatch, potentially leading to a rise in hs-cTnT levels in these patients.^4,10,11^ In this cohort we analyzed all consecutive stroke patients with imaging confirmed ischemic stroke and routine hs-cTnT measurements. However, with a median stroke severity of a NIHSS of four points, this represents a minor to moderate overall stroke severity. Given the observed association between stroke severity, large vessel occlusion and large territory infarctions with the presence of conditions triggering myocardial demand ischemia, more severely affected populations might even show an increased rate of such conditions.

Our analysis revealed that patients with a potential trigger of demand ischemia were older, more often female, and had higher rates of preexisting cardiovascular comorbidities. This is in line with the findings of several studies conducted in populations with MI showing that those of higher age, female sex, and with multiple comorbidities are at higher risk of developing type 2 MI.^1,9,10^ The development of cardiac complications after stroke including acute myocardial injury are known to be linked to higher mortality, longer hospitalization, and worse functional outcome.^6,18–20^ Therefore, our findings emphasize the importance of awareness of the treating physicians for increased cardiac stress in acute stroke patients. Several of the conditions facilitating myocardial demand ischemia could possibly be avoided or attenuated when detected and treated early. Timely diagnosis and prevention of blood pressure crises, respiratory failure or tachy-/bradyarrhythmia in patients with AF are just a few examples.

Our secondary objective was to assess the presence of triggers of demand ischemia in patients fulfilling the criteria of acute MI by retrospectively applying the criteria of the 4^th^ UDMI to our cohort.^1^ Among patients with acute MI, more than 60% had a trigger of myocardial demand ischemia (i.e. suspected type 2 MI). In all patients with acute MI and an evident trigger of demand ischemia undergoing coronary angiography, the diagnosis of type 2 MI was finally confirmed as no coronary culprit lesion was found. However, since only a minority of these patients (~30%) received invasive coronary angiography – given the setting of acute stroke – it cannot be ruled out that in some patients a concomitant atherothrombotic event had occurred (i.e. type 1 MI instead of type 2 MI). Generally, diagnosing type 2 MI can be challenging, as situations of myocardial oxygen supply/demand mismatch can evolve in many ways and are influenced by patients’ comorbidities.^1,10,12^ So far, no validated specific criteria or cut-offs for diagnosis of type 2 MI have been defined, leading to a very broad range of reported prevalences of type 2 MI in studies investigating patients with suspected MI.^10,21,22^

In this cohort, patients with evidence of acute MI according to 4^th^ UDMI showed significantly higher troponin values at baseline than those without. This is well in line with findings from the recent multicenter PRAISE study showing that elevated baseline hs-cTn levels of more than 5.5-times URL might help to differentiate stroke patients with and without acute MI.^8^ Of note, we observed a particularly strong difference regarding consecutive hs-cTn levels and absolute change in hs-cTn values between patients with and without acute MI. This underlines the importance of serial hs-cTn measurements in patients with acute ischemic stroke.

Strengths of this analysis are the prospective nature of the screening database and inclusion of all consecutive stroke patients with acute myocardial injury within the analyzed period. Furthermore, our study represents the first-time application of predefined cut-offs for conditions triggering myocardial oxygen supply/demand mismatch in a stroke population. We performed a thorough and comprehensive data collection for the assessment of triggers and diagnostic criteria for myocardial ischemia according to the 4th UDMI. Moreover, we precluded bias of age and sex by matching patients correspondingly.

Nevertheless, the following limitations must be considered when interpreting the results. First, although we used a prospective registry of routine clinical data, data regarding triggering factors of demand ischemia and diagnostic criteria for MI were collected retrospectively.^14^ Further cardiac imaging and invasive coronary angiography can be necessary to securely diagnose or rule out acute MI.^1^ Since especially invasive cardiac diagnostics was often not performed in this clinical setting of patients with acute stroke, the actual prevalence of acute MI might even be higher in stroke patients with acute myocardial injury. Second, we applied criteria to identify and validate the presence of triggers of myocardial oxygen supply/demand mismatch that were introduced in populations with cardiovascular disease.^11^ To our knowledge, this is the first time prevalence and types of these predefined potential triggers have systematically been assessed in patients with acute ischemic stroke. However, applying specific criteria for the diagnosis of type 2 MI has been controversially discussed, as the individual threshold for myocardial demand ischemia might be patient-specific.^1,10,12,23^ Nonetheless, no other validated criteria for diagnosis of type 2 MI exist so far and using specific cut-offs might provide an opportunity for objectifying the presence of potential triggers. On the other hand, the term “trigger” might imply a causal correlation. It must be emphasized that causality cannot be proven in our study. Depending on preexisting cardiovascular diseases and stroke severity, a given stroke patient might tolerate conditions of myocardial oxygen supply/demand mismatch in an individual manner with only some patients developing a myocardial infarction based on demand ischemia. Third, since acute myocardial injury is influenced by age and sex in stroke patients, we matched the control group to account for these factors. However, it is important to consider the resulting shift toward older age and presumably higher rate of cardiovascular disease burden when interpreting and generalizing the results, especially with regard to the overall rate of triggers identified in this cohort. Finally, this was a single-center study, and the majority of included patients had mild-to-moderate stroke. Future research in in larger and preferably multi-center cohorts that include different ranges of stroke severities is warranted to better understand the role of cardiac demand ischemia in the context of troponin elevation in acute ischemic stroke.

## Conclusions

This study reveals that myocardial demand ischemia may play an important role in the context of acute myocardial injury after stroke. Large strokes and those affecting the insular cortex were particularly common in patients with potential triggers of demand ischemia. Considering the established association between acute myocardial injury and higher short-term mortality as well as worse functional outcome after stroke, clinical awareness and early treatment of such triggering conditions might provide an opportunity for prevention of myocardial damage caused by demand ischemia.

Furthermore, stroke patients with acute myocardial injury and evidence of acute MI showed higher absolute troponin levels and more pronounced changes in repeated troponin measurements than those without acute MI. Absolute troponin levels and their dynamics as well as assessment of triggers of myocardial oxygen supply/demand-mismatch might guide stroke physicians in the decision-making process to identify patients that require timely invasive cardiac imaging and treatment.

## Supplementary Material

sj-docx-1-eso_23969873251346008

## Data Availability

The data supporting the study are available upon reasonable request.
