# Supplementary material for: Myocardial injury in patients with acute ischemic stroke: Prevalence and types of triggers of myocardial demand ischemia
Source: Eur Stroke J. 2026 Jan 1;11(1):23969873251346008. doi: 10.1093/esj/23969873251346008 (PMC12866251; doi:10.1093/esj/23969873251346008)
Supplement: sj-docx-1-eso_23969873251346008 [file sj-docx-1-eso_23969873251346008.docx]

**Myocardial injury in patients with ischemic stroke – prevalence and type of triggers of myocardial demand ischemia**

Supplemental material

**Supplemental table 1**: Predefined triggers of myocardial oxygen supply/demand mismatch for the analysis of acute stroke patients.

| Specific clinical standards for the definition of type 2 myocardial infarction; conditions reflecting an imbalance between myocardial oxygen supply and demand according to Saaby et al, 2013 | Adaptation of the criteria from Saaby et al for the analyzed neurologic cohort |
| --- | --- |
| Conditions with decreased oxygen supply: | |
| Anemia defined as a hemoglobin concentration <5.5 mmol/L for men and <5.0 mmol/L for women; | Anemia defined as a hemoglobin concentration <8 g/dl |
| Shock defined as systolic blood pressure <90 mm Hg together with signs of organ dysfunction (ie, metabolic acidosis, arterial oxygen tension <8 kPa, oliguria [diuresis <30 mL/h for at least 3 hours], or encephalopathy) | Shock defined as systolic blood pressure <90 mmHg or positive Shock-Index together with clinical symptoms and need of medical treatment |
| Bradyarrhythmia requiring medical treatment or cardiac pacing | Bradyarrhythmia requiring medical treatment or cardiac pacing |
| Coronary embolus in the presence of an increased risk of embolism (left heart endocarditis, intracardiac mural thrombus, documented venous thrombus, and a patent foramen ovale or atrial septum defect) | Coronary embolus in the presence of an increased risk of embolism (left heart endocarditis, intracardiac mural thrombus, documented venous thrombus, and a patent foramen ovale or atrial septum defect) |
| Respiratory failure with an arterial oxygen tension <8 kPa and clinical signs of acute respiratory failure lasting ≥20 minutes | Respiratory failure with an oxygen saturation <90% in pulse oximetry and need of medical treatment |
| Conditions with increased oxygen demand: | |
| Ventricular tachyarrhythmia lasting ≥20 minutes | Ventricular tachyarrhythmia lasting ≥20 minutes |
| Supraventricular tachyarrhythmia lasting ≥20 minutes with a ventricular rate >150 beats/min | Supraventricular tachyarrhythmia: heart rate >130 beats/min lasting ≥20 minutes with need of medical treatment |
| Hypertensive pulmonary edema defined as the presence of a systolic blood pressure >160 mmHg, signs of pulmonary edema, and a need for treatment with nitrates or diuretics | Hypertensive pulmonary edema defined as the presence of a systolic blood pressure >160 mmHg, signs of pulmonary edema, and a need for treatment with nitrates or diuretics |
| Arterial hypertension with a systolic blood pressure >160 mm Hg and concomitant left ventricular hypertrophy identified by echocardiography or electrocardiogram | Arterial hypertension with a systolic blood pressure >160 mm Hg and concomitant left ventricular hypertrophy identified by echocardiography or electrocardiogram |

Left: Criteria defined by Saaby et al. and right: adaptations made for the analysis of neurologic patients due to different measurement units of laboratory values, different availabilities of monitoring or diagnostic procedures, or differences in cohorts’ characteristics.

**Supplemental table 2**: Comparison of vital signs measured on stroke unit monitoring and prevalence of predefined trigger of myocardial oxygen supply/demand mismatch.

|  | **Total cohort**  **n= 508** | **Acute myocardial injury**  **n=254** | **No acute myocardial injury**  **n=254** | **p value** |
| --- | --- | --- | --- | --- |
| **Monitoring** |  |  |  |  |
| Highest systolic BP value, mmHg | 166 [152-182] | 170 [155-186] | 163 [149-177] | 0.001 |
| Lowest systolic BP value, mmHg | 113 [99-125] | 111 [96-125] | 114 [102-127] | 0.042 |
| Highest heart rate/min | 91 [79-110] | 99 [85-131] | 86 [76-97] | <0.001 |
| Lowest heart rate /min | 63 [54-75] | 64 [57-75] | 62 [56-71] | 0.034 |
| Lowest oxygen saturation in % | 93 [90-94] | 92 [89-94] | 93 [91-95] | 0.025 |
| Highest breathing rate/min | 23 [21-26] | 24 [22-27] | 23 [21-26] | <0.001 |
| Highest body temperature in C° | 37.1 [36.9-37.4] | 37.1 [36.8-37.4] | 37.1 [36.9-37.4] | 0.576 |
|  |  |  |  |  |
| **Trigger of oxygen demand/supply mismatch** |  |  |  |  |
| Presence of ≥1 trigger | 168 (33.1) | 107 (42.1) | 61 (24.0) | <0.001 |
| Multiple (≥2) triggers | 30 (5.9) | 23 (9.1) | 7 (2.8) | 0.003 |
| Anemia | 5 (1) | 4 (1.6) | 1 (0.4) |  |
| Respiratory failure | 83 (16.3) | 47 (18.5) | 36 (14.2) |  |
| Pulmonary edema | 3 (0.6) | 3 (1.2) | 0 (0) |  |
| Hypertension (>160mmHg) with LV hypertrophy | 74 (14.6) | 53 (20.9) | 21 (8.3) |  |
| Supraventricular tachyarrhythmia | 15 (3.0) | 12 (4.7) | 3 (1.2) |  |
| Shock | 18 (3.5) | 10 (3.9) | 8 (3.1) |  |
| Bradyarrhythmia | 1 (0.2) | 1 (0.4) | 0 (0.0) |  |

Values are presented as median with [interquartile range] and absolute numbers with (frequency %). Monitoring data and frequency of triggers of demand ischemia for the analyzed time period of interest from hospital admission to second hs-cTnT measurement. No patient fulfilled the cut-off for the predefined trigger ventricular tachycardia or cardiac embolism within the analyzed timeframe. BP: blood pressure, LV: left ventricular.
